# Supplementary material for: Self-reported high-risk behavior among first-time and repeat replacement blood donors; a four-year retrospective study of patterns
Source: PLoS One. 2024 Aug 8;19(8):e0308453. doi: 10.1371/journal.pone.0308453 (PMC11309432; doi:10.1371/journal.pone.0308453)
Supplement: Attachment — Submitted filename: Response to Reviewers.docx [file pone.0308453.s002.docx]

Felix Osei-Boakye

Department of Medical Laboratory Science
Sunyani Technical University
P. O. Box 206, Sunyani

Ghana

July 23, 2024

**THE EDITOR-IN-CHIEF**

**PLOS ONE**

Dear Editor,

**RESPONSE TO REVIEWERS**

Thank you for your email. I am pleased to resubmit our manuscript with ID: PONE-D-24-15733 titled **“Self-reported high-risk behavior among first-time and repeat replacement blood donors; a four-year retrospective study of patterns”** for your consideration.

The concerns raised by the editorial team have been addressed and highlighted below. Also, corrections have been made in the manuscript. I look forward to your favourable response.

Thank you.

Journal requirements:

When submitting your revision, we need you to address these additional requirements.

1. Please ensure that your manuscript meets PLOS ONE's style requirements, including those for file naming. The PLOS ONE style templates can be found at

https://journals.plos.org/plosone/s/file?id=wjVg/PLOSOne_formatting_sample_main_body.pdf and

<https://journals.plos.org/plosone/s/file?id=ba62/PLOSOne_formatting_sample_title_authors_affiliations.pdf>

The manuscript has been formatted according to the PLOS ONE style.

2. We note that your Data Availability Statement is currently as follows: [All relevant data are within the manuscript and its Supporting Information files.]

Please confirm at this time whether or not your submission contains all raw data required to replicate the results of your study. Authors must share the “minimal data set” for their submission. PLOS defines the minimal data set to consist of the data required to replicate all study findings reported in the article, as well as related metadata and methods (https://journals.plos.org/plosone/s/data-availability#loc-minimal-data-set-definition).

For example, authors should submit the following data:

- The values behind the means, standard deviations and other measures reported;

- The values used to build graphs;

- The points extracted from images for analysis.

Authors do not need to submit their entire data set if only a portion of the data was used in the reported study.

If your submission does not contain these data, please either upload them as Supporting Information files or deposit them to a stable, public repository and provide us with the relevant URLs, DOIs, or accession numbers. For a list of recommended repositories, please see https://journals.plos.org/plosone/s/recommended-repositories.

If there are ethical or legal restrictions on sharing a de-identified data set, please explain them in detail (e.g., data contain potentially sensitive information, data are owned by a third-party organization, etc.) and who has imposed them (e.g., an ethics committee). Please also provide contact information for a data access committee, ethics committee, or other institutional body to which data requests may be sent. If data are owned by a third party, please indicate how others may request data access.

All relevant data are within the manuscript and its Supporting Information files.

3. We note that Figure 1 in your submission contain [map/satellite] images which may be copyrighted. All PLOS content is published under the Creative Commons Attribution License (CC BY 4.0), which means that the manuscript, images, and Supporting Information files will be freely available online, and any third party is permitted to access, download, copy, distribute, and use these materials in any way, even commercially, with proper attribution. For these reasons, we cannot publish previously copyrighted maps or satellite images created using proprietary data, such as Google software (Google Maps, Street View, and Earth). For more information, see our copyright guidelines: http://journals.plos.org/plosone/s/licenses-and-copyright.

We require you to either (1) present written permission from the copyright holder to publish these figures specifically under the CC BY 4.0 license, or (2) remove the figures from your submission:

1. You may seek permission from the original copyright holder of Figure 1 to publish the content specifically under the CC BY 4.0 license.

We recommend that you contact the original copyright holder with the Content Permission Form (http://journals.plos.org/plosone/s/file?id=7c09/content-permission-form.pdf) and the following text: “I request permission for the open-access journal PLOS ONE to publish XXX under the Creative Commons Attribution License (CCAL) CC BY 4.0 (http://creativecommons.org/licenses/by/4.0/). Please be aware that this license allows unrestricted use and distribution, even commercially, by third parties. Please reply and provide explicit written permission to publish XXX under a CC BY license and complete the attached form.” Please upload the completed Content Permission Form or other proof of granted permissions as an "Other" file with your submission. In the figure caption of the copyrighted figure, please include the following text: “Reprinted from [ref] under a CC BY license, with permission from [name of publisher], original copyright [original copyright year].”

2. If you are unable to obtain permission from the original copyright holder to publish these figures under the CC BY 4.0 license or if the copyright holder’s requirements are incompatible with the CC BY 4.0 license, please either i) remove the figure or ii) supply a replacement figure that complies with the CC BY 4.0 license. Please check copyright information on all replacement figures and update the figure caption with source information. If applicable, please specify in the figure caption text when a figure is similar but not identical to the original image and is therefore for illustrative purposes only.

The following resources for replacing copyrighted map figures may be helpful:

USGS National Map Viewer (public domain): http://viewer.nationalmap.gov/viewer/

The Gateway to Astronaut Photography of Earth (public domain): http://eol.jsc.nasa.gov/sseop/clickmap/

Maps at the CIA (public domain): https://www.cia.gov/library/publications/the-world-factbook/index.html and

<https://www.cia.gov/library/publications/cia-maps-publications/index.html>

NASA Earth Observatory (public domain): http://earthobservatory.nasa.gov/

Landsat: <http://landsat.visibleearth.nasa.gov/>

USGS EROS (Earth Resources Observatory and Science (EROS) Center) (public domain): http://eros.usgs.gov/#

Natural Earth (public domain): <http://www.naturalearthdata.com/>

Figure 1 has been removed from the manuscript.

4. Please review your reference list to ensure that it is complete and correct. If you have cited papers that have been retracted, please include the rationale for doing so in the manuscript text, or remove these references and replace them with relevant current references. Any changes to the reference list should be mentioned in the rebuttal letter that accompanies your revised manuscript. If you need to cite a retracted article, indicate the article’s retracted status in the References list and also include a citation and full reference for the retraction notice.

The references have been updated.

**Reviewer #1:**

1. The manuscript has the information but the way it was presented could have been better. The title 'Study design and setting' has the year the study was commenced omitted making it look like the study was conducted only in 2024. This column should be re-written for fluency.

In this study, we retrospectively reviewed and collected archived data spanning 2017 and 2020. And, this was done as a onetime sampling in 2024 (from 14^th^ March to 4^th^ April 2024). So, for the purpose of clarity, the study was conducted only in 2024, using archived/retrospective data. This information has been modified and the clarity has been improved. This can be found on page 6, line 101-104.

2. Check and correct dates throughout the text.

All the dates have been reformatted.

3. Under the heading study population. Family replacement donors undergo screening for blood donation not transfusion as stated since they are the donors not patients.

The sentence has been corrected.

4. The heading pre- screening of blood donors and administration of questionnaire made the study look like a prospective rather than a retrospective study. This column can be removed, and the information summarized into the data collection column and stated retrospectively.

The section has been removed and the necessary changes have been effected.

**Reviewer #2:**

1. Why only Tuberculosis is add in the high risk behaviour other than STD's? Why not other infections?

History of sexually transmitted infections have been reported in the lower part of both tables 2 and 3 on pages 12 and 14, respectively and figure 2 on page 16.

- Have you tested positive for gonorrhea or other STIs?

- Do you or your partner have HIV or hepatitis?

2. Why no association of Haemoglobin has been seen any where? I think you should also give the haemoglobin of those who had STD's

There was no significant association between haemoglobin and sexually transmitted infections. This has been included in the revised manuscript as Figure 2, on page 16, line 302-318.

3. STD's are given generally, why not specific STD is mentioned during history taking?

The retrospective nature of the study did not permit modification of the questions and or decoupling of the STIs. This has been captured as a limitation to the study, on page 21, line 426-428.

4. Were female donors asked properly about STD's history? Were female phlebotomist/ doctors available for their history?

This was a retrospective study, and only data from archived records were accessible. Consequently, the researchers did not play any role in the administration of the questionnaires.

5. Which guidelines are followed at GHANA for UDHQ? As per AABB, if tattoo is now done by a closed system, donor is accepted
   Is there any criteria of reentry for such donors specially repeat donors?

The document used in Ghanaian blood centers that is similar to the UHDQ is the ‘Donor Clinical Record’. This is available at <https://figshare.com/s/69f8efdc1ddaf1ec6dd5>.

Donors with tattoos are permitted to donate blood after six months, if they test negative for STIs, and also, satisfy other pre-screening conditions. This is because hepatitis B virus which is among the infections screened for has an incubation period of about six months.
